# Low-density lipoprotein cholesterol and radiotherapy-induced carotid atherosclerosis in subjects with head and neck cancer

**DOI:** 10.1186/1748-717X-9-134

**Published:** 2014-06-11

**Authors:** Eduardo B Pereira, Tiago Gemignani, Andrei C Sposito, José R Matos-Souza, Wilson Nadruz Jr

**Affiliations:** 1 Departamento de Clínica Médica, Faculdade de Ciências Médicas, Universidade Estadual de Campinas, Cidade Universitária “Zeferino Vaz”, Campinas, SP 13081-970, Brasil

**Keywords:** Carotid artery, Intima-media thickness, Head and neck cancer, Radiotherapy, Low-density lipoprotein cholesterol

## Abstract

**Background:**

Radiotherapy (RT) is a risk factor for accelerated carotid artery atherosclerotic disease in subjects with head and neck cancer. However, the risk factors of RT-induced carotid artery remodeling are not established. This study aimed to investigate the effects of RT on carotid and popliteal arteries in subjects with head and neck cancer and to evaluate the relationship between baseline clinical and laboratory features and the progression of RT-induced atherosclerosis.

**Findings:**

Eleven men (age = 57.9 ± 6.2years) with head and neck cancer who underwent cervical bilateral irradiation were prospectively examined by clinical and laboratory analysis and by carotid and popliteal ultrasound before and after treatment (mean interval between the end of RT and the post-RT assessment = 181 ± 47 days). No studied subject used hypocholesterolemic medications. Significant increases in carotid intima-media thickness (IMT) (0.95 ± 0.08 vs. 0.87 ± 0.05 mm; p < 0.0001) and carotid IMT/diameter ratio (0.138 ± 0.013 vs. 0.129 ± 0.014; p = 0.001) were observed after RT, while no changes in popliteal structural features were detected. In addition, baseline low-density lipoprotein cholesterol levels showed a direct correlation with RT-induced carotid IMT change (r = 0.66; p = 0.027), while no other studied variable exhibited a significant relationship with carotid IMT change.

**Conclusions:**

These results indicate that RT-induced atherosclerosis is limited to the irradiated area and also suggest that it may be predicted by low-density lipoprotein cholesterol levels in subjects with head and neck cancer.

## Background

Radiotherapy (RT) is a risk factor for stroke in patients treated for head and neck cancer [1] and the major underlying mechanism of this association is the development of carotid artery atherosclerotic disease secondary to local radiation exposition [1,2]. However, the risk factors of carotid artery remodeling after head and neck RT are not established. Traditional vascular risk factors, such as diabetes mellitus, hypertension, and smoking have been extensively reported to exert no major influence on the development of irradiation-induced carotid remodeling [3-5], while the influence of radiation dose remains controversial [6]. Conversely, some animal work and one case–control study in humans have suggested that hypercholesterolemia might contribute to the development of atherosclerosis in irradiated arteries [7-9]. Nevertheless, no prospective study has evaluated whether lipid levels are indeed associated with RT-induced carotid remodeling.

Although RT-induced atherosclerosis is assumed to be limited to the irradiated area [6], previous data indicated that RT might change circulating levels of pro-atherogenic molecules [10]. In addition, subjects with cancer have been reported to present accelerated atherosclerosis even in the absence of radiation exposition [11]. This body of evidence raises the hypothesis that arterial remodeling could be not restricted to irradiated areas in subjects with cancer.

To our knowledge, only 4 prospective studies evaluated the development of RT-induced carotid atherosclerosis in subjects with head and neck cancer [5,10,12,13]. These reports showed increases in carotid atherosclerosis from 3 months up to 3 years after the end of RT sessions. Nevertheless, none of these studies evaluated the remodeling of remote arteries or disclosed the risk factors that might contribute to the development of carotid artery atherosclerosis. Therefore, the aims of this study were to prospectively investigate the effects of RT on carotid and popliteal arteries in subjects with head and neck cancer and to evaluate the correlation between baseline clinical and laboratory features and the progression of RT-induced atherosclerosis.

## Findings

### Methods

Eleven consecutive patients with head and neck cancer from the Radiotherapy Sector of the Clinics Hospital of the University of Campinas were prospectively evaluated in this study. Inclusion criteria were planned head and neck radiotherapy, male gender and age between 40 and 70years old. Exclusion criteria were infectious, inflammatory or auto-immune active diseases and acute coronary syndromes or stroke in the last year and use of hypocholesterolemic medications (statins, fibrates or ezetimibe). The primary cancer sites were nasopharynx (n = 1), larynx (n = 3), oropharynx (n = 2) and oral cavity (n = 5). This study was approved by the Human Research Ethics Committee of the University of Campinas. All subjects gave written informed consent to participate.

Clinical and vascular measurements and blood collection for laboratory analysis were performed at two-time points: (1) at the inclusion in the study, prior to radiotherapy (pre-RT) and (2) between 4 to 9 months after radiotherapy sessions were completed (post-RT). The mean interval between the end of RT and the post-RT assessment was of 181 ± 47 days. Body mass index was calculated as body weight divided by height squared. Blood pressure was measured using a validated digital oscillometric device (HEM-705CP; Omron Healthcare, Japan). Complete blood count, fasting low-density lipoprotein cholesterol, high-density lipoprotein cholesterol, triglycerides, creatinine and glucose were measured using standard laboratory techniques.

All subjects required bilateral cervical irradiation, with a radiation field that included both carotid arteries. All patients were treated by cobalt therapy, fraction size of 2 Gy per daily fraction with five fractions per week (Cobalt unity, Alcyon II, General Electric, Milwaukee, WI, USA). Patients were treated with RT portals using parallel-opposed fields encompassing the primary, draining sites, and bilateral necks, treated to 44 Gy in 2 Gy daily fractions in 4.5 weeks. During the last 2–2.5 weeks, smaller portals using parallel-opposed fields boost to all gross disease were treated to 26 Gy. All patients received a low anterior neck field matched to upper neck RT fields. In general, 70 Gy was delivered in 2 Gy daily fractions, with five fractions per week, during 7 weeks.

Common carotid ultrasonography was performed by a single physician using a Vivid 3 Pro apparatus (General Electric, Milwaukee, WI, USA) equipped with a 10-MHz linear-array transducer as previously described [14,15]. Measurements of carotid artery intima-media thickness (IMT) were done at 2 cm proximal to the carotid, while popliteal artery IMT was measured at 1 cm distal to the emergence of the geniculate artery. Diastolic and systolic internal diameters were obtained by continuous tracing of the intimal-luminal interface of the near and far walls of the studied arteries in 3 cycles and averaged. Artery IMT and diameters were always obtained in plaque-free areas and were calculated as the average from both right and left arteries measurements. Intraobserver and interobserver carotid and popliteal IMT and diameters variabilities were <5%.

Based on a previous study [10], a sample size of at least 10 individuals was considered suitable to detect significant differences in carotid IMT between pre-RT and post-RT time points. Data were presented as mean ± SD for continuous variables. The Kolmogorov–Smirnov test was used to test for normal distribution of the variables. Paired *t*-test and chi-square were used to compare the studied variables before and after radiotherapy. Bivariate correlation analysis was assessed by the Pearson’s Method. A two-sided p-value of 0.05 was considered statistically significant.

### Results

**Figure 1:**
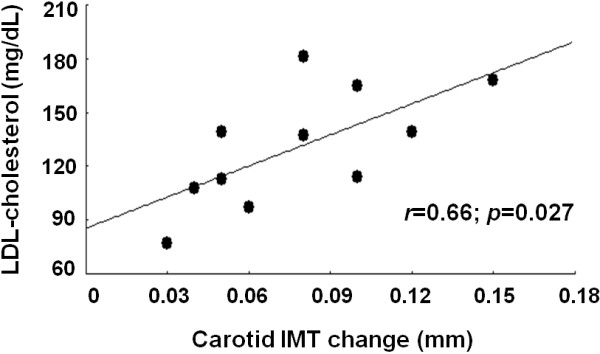
Bivariate correlation between baseline low-density lipoprotein cholesterol (LDL-cholesterol) levels and radiotherapy-induced carotid intima-media thickness (IMT) change.

Clinical and laboratory features of studied subjects before and after RT sessions are shown in Table 1. No differences in clinical and laboratory characteristics were detected after RT in comparison with the pre-RT time point. The vascular features of studied subjects are shown in Table 2. Carotid IMT and carotid IMT/diameter ratio showed significant increases after RT, while no changes in carotid diameters or in any popliteal variable were detected after radiation therapy. In addition, 1 (9%) and 2 (18%) subjects presented popliteal and carotid plaques before RT, respectively, while 1 (9%) and 4 (36%) subjects presented popliteal and carotid plaques after RT. However, the differences in carotid plaques prevalence before and after RT did not reach statistical significance.In order to investigate potential predictors of RT-induced carotid atherosclerosis, bivariate correlation analysis between carotid IMT change (the difference between post-RT and pre-RT carotid IMT measurements) and baseline clinical and laboratory features was performed. Baseline low-density lipoprotein cholesterol levels showed a direct correlation with carotid IMT change (r = 0.66; p-0.027) (Figure 1), while no other clinical and laboratory variable, including the interval between the end of RT and the post-RT assessment, exhibited a significant correlation with carotid IMT change.

**Table 1:** Clinical and laboratory features of studied subjects

	**Pre-RT**	**Post-RT**	p*
Age, years	57.9 ± 6.2		
Hypertension, n (%)	1 (9)		
Diabetes mellitus, n (%)	1 (9)		
History of smoking, n (%)	11 (100)		
Smoking, pack-years	59 ± 48		
Body mass index, kg/m^2^	21.8 ± 6.2	21.6 ± 3.2	0.753
Systolic blood pressure, mmHg	110 ± 12	103 ± 11	0.104
Diastolic blood pressure, mmHg	69 ± 9	66 ± 8	0.437
Glycemia, mg/dL	86 ± 12	88 ± 12	0.456
Triglycerides, mg/dL	89 ± 27	117 ± 53	0.077
HDL-cholesterol, mg/dL	50 ± 14	49 ± 15	0.978
LDL-cholesterol, mg/dL	131 ± 32	146 ± 32	0.118
Creatinine, mg/dL	0.88 ± 0.20	0.98 ± 0.37	0.157
Hemoglobin, g/dL	13.1 ± 1.6	12.5 ± 2.3	0.476
Leucocytes, /mm^3^	8.85 ± 4.11	7.12 ± 3.03	0.242
Platelets, /mm^3^	304 ± 106	264 ± 112	0.159

Legend. RT – radiotherapy; HDL – high-density lipoprotein; LDL – low-density lipoprotein; *pre-RT vs. post-RT.

**Table 2:** Vascular features of studied subjects

	**Pre-RT**	**Post-RT**	p*
*Common carotid artery features*			
IMT, mm	0.87 ± 0.05	0.95 ± 0.08	<0.0001
Systolic diameter, mm	7.08 ± 0.86	7.24 ± 0.77	0.080
Diastolic diameter, mm	6.80 ± 0.85	6.91 ± 0.75	0.115
IMT/diastolic diameter ratio	0.129 ± 0.014	0.138 ± 0.013	0.001
*Popliteal artery features*			
IMT, mm	0.93 ± 0.04	0.94 ± 0.03	0.271
Systolic diameter, mm	5.51 ± 0.50	5.61 ± 0.46	0.101
Diastolic diameter, mm	5.26 ± 0.50	5.32 ± 0.43	0.353
IMT/diastolic diameter ratio	0.179 ± 0.018	0.177 ± 0.015	0.263

Legend. RT – radiotherapy; IMT – intima-media thickness; *pre-RT vs. post-RT.

## Discussion

A major finding of the present report was the significant correlation between baseline low-density lipoprotein cholesterol levels and carotid IMT change, which indicates that lipid levels may predict the development of RT-induced carotid atherosclerosis. Likewise, animal studies have shown that elevated serum cholesterol contributes to the development of radiation-induced carotid artery stenosis [7,8], while an association between hypercholesterolemia/hyperlipidemia and a faster progression of irradiation-induced carotid atherosclerosis in humans was reported in a case–control study [9]. Curiously, the 4 prospective studies that evaluated the effects of RT on carotid remodeling in patients with head and neck cancer did not investigate the role of lipid levels in this regard [5,10,12,13]. Thus, our findings seem to sound clinically relevant since they provide the first prospective data showing that low-density lipoprotein cholesterol levels might be a predictor of RT-induced carotid atherosclerosis. Furthermore, they support the need for additional studies evaluating whether blood low-density lipoprotein cholesterol lowering should be initiated before cervical irradiation in subjects with head and neck cancer.

RT was shown to be associated with changes in circulating levels of pro-atherogenic molecules [10] and subjects with cancer were reported to develop accelerated atherosclerosis even in the absence of radiation exposition [11]. Based on these evidences, one hypothesis of the present study was that RT could influence vascular remodeling not only in local but also in remote vessels. In order to address this issue, the features of popliteal arteries of the studied patients were evaluated before and after RT, but no changes in popliteal IMT, diameter and IMT/diameter ratio were detected. Although it can be argued that the time of follow-up in our study was not sufficient to detect significant changes in popliteal artery structure, the present findings agree with data from other report which showed no changes in carotid IMT of subjects with prostate cancer treated with RT [10], thus strengthening the notion that RT-induced vascular remodeling is limited to local irradiated vessels.

A potential limitation of our study was the relative small sample size. Furthermore, the inclusion of only subjects with history of smoking means that the results cannot yet be applied to non-smokers.

## Conclusion

The present report showed that cervical irradiation is associated with early increases in carotid, but not in popliteal atherosclerosis and that low-density lipoprotein cholesterol levels may be a predictor of RT-induced carotid IMT increases in patients with head and neck cancer. Further studies are necessary to evaluate whether blood low-density lipoprotein cholesterol lowering should be initiated before cervical irradiation in this population in order to modify the development of RT-induced atherosclerosis.

## Abbreviations

IMT: Intima-media thickness; RT: Radiotherapy.

## Competing interests

All authors declare that they have no competing interests.

## Authors’ contributions

EBP and WNJ designed research; EBP, TG, JRMS conducted research; ACS, JRMS and WNJ analyzed data; EB, ACS and WNJ wrote paper. All authors read and approved the final manuscript.
